# Proximate Composition, Physicochemical, Functional, and Antioxidant Properties of Flours from Selected Cassava (*Manihot esculenta* Crantz) Varieties

**DOI:** 10.1155/2021/6064545

**Published:** 2021-12-08

**Authors:** R. A. T. Nilusha, J. M. J. K. Jayasinghe, O. D. A. N. Perera, P. I. P. Perera, C. V. L. Jayasinghe

**Affiliations:** ^1^Department of Food Science and Technology, Faculty of Applied Sciences, University of Sri Jayewardenepura, Gangodawila, Nugegoda, Sri Lanka; ^2^Department of Food Science and Technology, Faculty of Livestock, Fisheries and Nutrition, Wayamba University of Sri Lanka, Makandura, Gonawila, Sri Lanka; ^3^Department of Horticulture and Landscape Gardening, Faculty of Agriculture and Plantation Management, Wayamba University of Sri Lanka, Makandura, Gonawila, Sri Lanka

## Abstract

Cassava flour has a high potential to contribute as a raw material in the food industry. This study was aimed at characterizing flours from Sri Lankan cassava varieties with a view to explore the potential in food applications. Flours prepared from five cassava varieties, namely, *Kirikawadi*, MU51, *Swarna*, *Shani*, and *Suranimala*, were analyzed for proximate composition and physicochemical, functional, and antioxidant properties using standard methods. Flours from tested cassava varieties contained <1% crude fat and <2% crude protein. Flour from MU51 contained the highest amount of HCN (48.05 mg/kg) while flour from *Suranimala* contained the lowest (4.85 mg/kg). Total starch and amylose contents of flours were significantly lower (*p* < 0.05) than those of commercial wheat flour. Flour from *Suranimala* contained approximately similar amylopectin content as commercial wheat flour. Water absorption capacity, oil absorption capacity, water solubility index, swelling power, emulsion activity, and emulsion stability of flours from five cassava varieties were significantly higher (*p* < 0.05) than those of commercial wheat flour. *Swarna* was identified as the richest source of phenolic compounds (4.44 mmol GAE/100 g dry weight) among the five varieties. Results showed the promising application potential of flours from these five cassava varieties in different food applications such as weaning foods, bakery foods, and edible films.

## 1. Introduction

Cassava (*Manihot esculenta* Crantz) is an important perpetual root crop that provides food for over 500 million people in the world [1, 2]. It is a consistent and inexpensive food source assuring the global food security by providing the carbohydrate dietary requirement of the people especially, in the low-income countries [3]. Cassava has many applications in the product diversification, varying from food to nonfood products [4]. Being a rich source of carbohydrate, cassava facilitates the utilization of its flour in the applications such as manufacturing cassava strips [5], short biscuits [6], pasta [7, 8], porridges [9], instant flour [10], and starch films [11].

Cassava storage roots have a short shelf life due to postharvest physiological deterioration that occurs shortly after harvesting [12]. It causes discoloration of the roots making them unsuitable for consumption or being used as a raw material in the food industry. Moreover, the presence of hydrogen cyanide (HCN) in roots limits its usage in the food industry. Proper storage conditions have not yet been developed to overcome the high postharvest losses of cassava roots. Immediate channeling of the harvested storage roots to form value-added materials is worthwhile to consider it as one of the ways for maximizing its utilization.

Although there have been available data on characteristics of cassava flour worldwide, there is insufficient information about Sri Lankan cassava varieties. Being a tropical country, the climatic conditions in Sri Lanka are well suited for cassava cultivation [13]. Characterization of the properties of cassava flour is of the utmost importance for ensuring its efficient utilization in diversified purposes. Thus, characterization of cassava flour derived from five Sri Lankan varieties can be the basis for understanding and expanding the knowledge of how to enhance their utilization as a value-added raw material in the food processing industry.

## 2. Materials and Methods

### 2.1. Materials

Mature roots of five cassava varieties, *Kirikawadi*, MU51, *Swarna*, *Shani*, and *Suranimala*, grown under the same soil and climatic conditions at the Horticultural Crop Research and Development Institute, Department of Agriculture, Gannoruwa, Peradeniya, Sri Lanka, were used for the study. Commercially available wheat flour was used as the control flour sample. All the chemicals used were of analytical grade.

### 2.2. Preparation of Cassava Flour

All five cassava varieties were cleaned and peeled. Cassava slices of 1 mm thickness were obtained from the flesh and dipped in water (water : slices; 3 : 1 *w*/*w*) for 24 h at 30°C [14]. Subsequently, cassava slices were washed twice with potable water and dehydrated in a food dehydrator (IFTD, India) at 50°C for 24 h. Dehydrated cassava slices were ground in a household grinder (Pansonic-MX-GX1511W, Japan) and screened through a 0.425 mm sieve. The flour was vacuum-packed and kept at room temperature (~30°C) for further analysis.

### 2.3. Determination of Proximate Composition

For the proximate analysis, the methodologies outlined in AOAC [15] were used. The moisture and ash contents of flour were determined using the gravimetric principle, the oven drying and dry ashing methods, respectively. A Soxhlet extraction method was used to determine the crude fat content of flours. A micro-Kjeldahl method was used to determine the crude protein contents. Total carbohydrate contents of the flour samples were determined by subtracting the sum of the values of crude protein, crude fat, and ash contents (% wet basis) of the sample from 100 [16].

### 2.4. Determination of Physicochemical Properties

#### 2.4.1. Hydrogen Cyanide (HCN) Content

The HCN content of flours was determined by an in-house method of Bureau Veritas, Sri Lanka.

#### 2.4.2. Color

The color was measured as lightness (*L*∗), redness/greenness (*a*∗), and yellowness/blueness (*b*∗) using a Chroma meter (LOVIBOND® LC 100, England).

#### 2.4.3. Total Starch Content

Total starch content was determined using the method described by Alamu et al. [17]. A 0.2 mg of flour was mixed with 96% ethanol (1 mL), 96% hot (40°C) ethanol (10 mL), and distilled water (2 mL). After that, the centrifugation was done for 10 min at 2,000 rpm. The sediment was mixed with 70% HClO_4_ (7.5 mL) and kept for 1 h and vortexed with distilled water (17.5 mL). After that, C_6_H_6_O (0.5 mL), conc. H_2_SO_4_ (2.5 mL), and distilled water (0.95 mL) were added and vortexed. The absorbance was measured at room temperature with a UV-Vis spectrophotometer (SELECTA 4120025, Spain) at 490 nm.

#### 2.4.4. Starch Granule Size and Shape

The size and shape of the starch granules were determined by using a scanning electron microscope (SEM) (EVO LS 15, Canada).

#### 2.4.5. Amylose and Amylopectin Contents

Amylose content was determined by using the method described by Zhou et al. [18]. A 20 mg of flour was weighed and dissolved in 85% methanol (5 mL) with gradual mixing for 30 min at 60°C. Then, the centrifugation was done at 2000 rpm, and the supernatant was discarded. Extraction was repeated for three times, and the lipid free sediment was obtained. Lipid-free sediment was mixed with distilled water (1 mL) and 1 M NaOH (2 mL). It was incubated for 30 min in a water bath (MEMMERT WNB14, India) at 95°C with gradual mixing. Solubilized sediment (0.1 mL) was added to 0.5% C_2_HCl_3_O_2_ (5 mL) separately, and 0.01 N KI (0.05 mL) was added to the solution. Blue color was read at 620 nm after 30 min, with the UV-Vis spectrophotometer (SELECTA 4120025, Spain). Potato amylose was used to develop the standard curve. Amylopectin content was expressed as a percentage as the deduction of amylose content of the sample from the total starch content of the same sample.

### 2.5. Determination of Functional Properties

#### 2.5.1. Water and Oil Absorption Capacities (WAC and OAC)

The WAC and OAC were calculated using the approach given by Sosulski et al. [19]. One gram of flour was combined with distilled water (10 mL; refined soy bean oil for OAC) and incubated at 30°C for 30 min. After that, the centrifugation was done at 3000 rpm for 30 min. The sediment's weight was calculated. The WAC and OAC were determined as a percentage of the wet weight of the flour according to

(1) $$\begin{array}{c} \%WAC=\frac{\text{Weight of the water added to the sample}–\text{Weight of the water removed from the sample}}{\text{Weight of the flour sample}}\times 100, \end{array}$$

(2) $$\begin{array}{c} \%OAC=\frac{\text{Weight of the oil added to the sample}–\text{Weight of the oil removed from the sample}}{\text{Weight of the flour sample}}\times 100. \end{array}$$

#### 2.5.2. Water Solubility Index (WSI) and Swelling Power (SP)

The WSI and SP were calculated using the approach given by Leach et al. [20]. One gram flour was dissolved in distilled water (10 mL) in a graduated centrifuged tube. The mixture was heated at 85°C in a water bath (MEMMERT WNB14, India) for 30 min with gentle mixing. Then, it was cooled to room temperature, and the centrifugation was done at 2200 rpm for 15 min. The solubility was determined by evaporating the supernatant in a drying oven (SELECTA 64.P, Netherlands) at 105°C. The sediment paste was weighed. WSI and SP were calculated according to5

(3) $$\begin{array}{c} \%WSI=\frac{\text{Weight of the soluble}}{\text{Weight of the flour sample}}\times 100, \end{array}$$

(4) $$\begin{array}{c} SP=\frac{\text{Weight of the sediment}}{\text{Weight of the flour sample}\times \left(100-\text{WSI}\right)}. \end{array}$$

#### 2.5.3. Gelatinization Temperature (GT)

The GT was determined as the method described by Chandra et al. [21]. One gram of flour was weighed, and distilled water (10 mL) was added. Until a solid gel is formed, the mixture was incubated in a water bath (MEMMERT WNB14, India). The temperature, which plays a role in the gel formation, was measured as the GT.

#### 2.5.4. Bulk Density (BD)

The BD was calculated using the approach given by Chandra et al. [21]. The volume of flour was measured as the BD by putting 20 g of flour into a 250 mL measuring cylinder.

#### 2.5.5. Emulsion Activity (EA) and Emulsion Stability (ES)

The EA and ES were calculated using the approach given by Chandra et al. [21]. In a centrifuge tube, one gram of flour was combined with distilled water (5 mL) and soybean oil (5 mL). It was centrifuged for 5 min at 3000 rpm. The percentage of the height of the emulsion layer to the height of the mixture was measured as EA. After incubating the emulsion in a water bath (MEMMERT WNB14, India) at 80°C for 30 min, the ES was measured. The temperature was lowered for 15 min under running water before being centrifuged for 15 min at 3000 rpm. The ES was calculated as the ratio of the height of the emulsified layer to the total height of the mixture.

### 2.6. Determination of Antioxidant Properties

#### 2.6.1. Extraction of Phenolic Compounds

Extraction of the phenolic compounds of the flour was done according to the method described by Chandrasekara and Kumar [22]. Flour (1 g) was dissolved in 80% methanol (10 mL) and was incubated in a water bath (MEMMERT WNB14, India) at 50°C for 40 min. Then, the centrifugation was done at 4000 rpm, and the supernatant was transferred into a volumetric flask (50 mL). The extraction was repeated using another 80% methanol (10 mL), and the supernatant was transferred into the same volumetric flask (50 mL) and marked up to 50 mL using 80% methanol. The filtrate was stored in a dark colored tightly closed bottle and kept under frozen condition (-18°C).

#### 2.6.2. Total Phenolic Content (TPC)

The TPC was determined as the method described by Singleton et al. [23]. The extraction in methanol (1 mL) was added into a volumetric flask (25 mL), containing distilled water (9 mL). Then, the Folin-Ciocalteu reagent (1 mL) was added. After 5 min, 7% Na_2_CO_3_ (10 mL) was added. It was diluted up to the 25 mL with distilled water. After incubation for 90 min at 30°C, the measurement of absorbance was done at 750 nm with the UV-Vis spectrophotometer (SELECTA 4120025, Spain). The TPC was expressed as mmol gallic acid equivalents (GAE)/100 g dry weight.

#### 2.6.3. Total Flavonoid Content (TFC)

The TFC was determined according to the AlCl_3_ colorimetric method as described by Kalita et al. [24]. The standard curve was developed using quercetin. For the stock solution (0.5 mL) of each extract, methanol (1.5 mL), 1% AlCl_3_ (0.1 mL), 1 M CH_3_CO_2_K (0.1 mL), and distilled water (2.8 mL) were added. The measurement of absorbance was done at 415 nm with the UV-Vis spectrophotometer (SELECTA 4120025, Spain). The TFC was measured as mmol quercetin equivalents (QE)/100 g dry weight.

#### 2.6.4. 1,1-Diphenyl-2-picrylhydrzyl (DPPH) Radical Scavenging Activity (DRSA)

Antioxidant activity was determined according to Kourouma et al. [25] with slight modifications. The methanol extraction of flour (1.5 mL) was mixed with DPPH solution (1.5 mL). After 30 min of incubation in a dark place at 30°C, the absorbance was measured at 517 nm using the UV-Vis spectrophotometer (SELECTA 4120025, Spain). The percentage inhibition of absorbance was measured by the absorbance of control over the absorbance difference of the control and sample into percentage.

#### 2.6.5. Ferric Reducing Antioxidant Power (FRAP)

The FRAP was determined according to the method of Davies-Hoes et al. [26]. The methanol extract (1 mL) was mixed with 0.2 M phosphate buffer (2.5 mL; pH 6.6) and 1% (*w*/*v*) of C_6_N_6_FeK_3_ (2.5 mL). After that, the incubation of the mixture was done in a water bath (MEMMERT WNB14, India) at 50°C for 20 min, and then, 10% (*w*/*v*) C_2_HCl_3_O_2_ (2.5 mL) was added and the centrifugation was done for 10 min at 3000 rpm. The supernatant (2.5 mL) was added to distilled water (2.5 mL) and 0.1% (*w*/*v*) FeCl_3_ (0.5 mL). Absorbance was measured using the UV-Vis spectrophotometer (SELECTA 4120025, Spain) at 700 nm.

### 2.7. Statistical Analysis

All experiments were conducted in triplicate, and the data were expressed as mean ± standard deviation. The sample means were compared at the 95% confidence level (*p* < 0.05) using Tukey's test in SPSS 16.0 software.

## 3. Results and Discussion

### 3.1. Morphology of Selected Cassava Varieties

**Figure 1:**
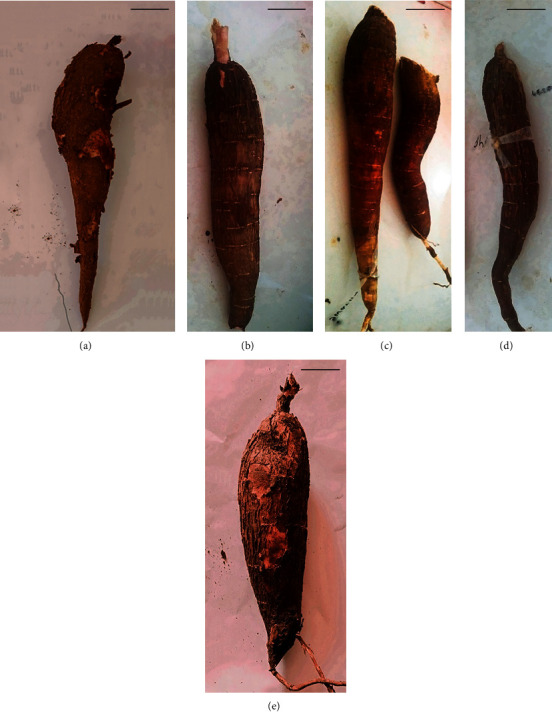
Mature roots of selected Sri Lankan cassava varieties: (a) *Kirikawadi*, (b) MU51, (c) *Swarna*, (d) *Shani*, and (e) *Suranimala*. Scale bar = 2 cm.

**Figure 2:**
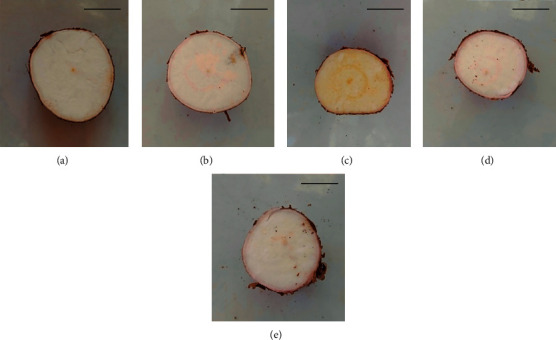
Cross sections of mature roots of selected Sri Lankan cassava varieties: (a) *Kirikawadi*, (b) MU51, (c) *Swarna*, (d) *Shani*, and (e) *Suranimala*. Scale bar = 20 mm.

The shape and size of cassava roots are important morphological characteristics, determined by the variety, growing conditions, and harvesting time. The studied mature roots of five varieties had different sizes (Figure 1). The yield of five varieties is reported in the range of 22-75 t/ha [27]. The color of the outer and inner layers of mature *Kirikawadi* roots was white. However, MU51, *Shani*, *Suranimala*, and *Swarna* roots had a brown color outer layer and pink color inner layer (Figure 1). The flesh of *Kirikawadi*, MU51, *Shani*, and *Suranimala* was white while the flesh of *Swarna* was light yellow reflecting the differences in chemical composition (Figure 2).

### 3.2. Proximate Composition

The overall chemical composition of studied cassava flour samples was compared with that of commercial wheat flour (Table 1). The total carbohydrate is the major component in cassava flour (>80%) as shown in the data. It was ranged from 86.28% (*Swarna*) to 93.13% (MU51) and higher than the carbohydrate content of wheat flour (Table 1). Approximately, similar carbohydrate contents in cassava flour have been observed by Tambo et al. [28] and Dudu et al. [4] which were 96.95% and 83.55%, respectively. Due to high amount of carbohydrate contents (>80%) of the flours of studied cassava varieties, it can be utilized in formulating composite flour blends in the food manufacturing. In particular, high carbohydrate contents facilitate manufacturing foods such as cassava strips [5], snacks [29], gruels [27, 30], short biscuits [6], porridges [9], instant flours [10], and gluten-free pasta [8].

Moisture content is an important parameter when determining the shelf life of foods. It was found that MU51 cassava flour had the lowest moisture content (4.45%) when compared with the commercial wheat flour (12.49%). It should be noted that moisture contents of cassava flours were varied with the variety. Results revealed that the moisture contents of all flour samples were less than the recommended moisture content (13% *W*/*W*) [31] of edible cassava flour. Low moisture contents of flours have appropriate shelf life stability when stored in packaging materials with proper moisture barrier properties [32]. Moisture contents of studied cassava flour samples were deviated from the moisture contents of cassava flour obtained by Tambo et al. [28] (12.55%) and Klang et al. [30] (13.86%). This might be due to the climatic conditions, variety, and processing differences.

The ash content reflects the inorganic mineral content of flour samples. As shown in Table 1, ash contents are comparatively similar among five cassava varieties. These findings are similar to those of Tambo et al. [28] and Dudu et al. [4] who observed 1.05% and 1.64% of ash contents, respectively. Moreover, ash contents of cassava flour were similar to ash content of commercial wheat flour. Therefore, substitution of wheat flour with studied cassava flour will give a similar nutritional profile in the case of mineral elements.

Crude fat contents of studied cassava flour are lower than 1% (Table 1). According to the results, the highest fat contents were observed in *Shani* (0.63%) and *Suranimala* (0.64%) varieties which were similar to the observations of Tambo et al. [28] who observed 0.63% fat content from a cassava flour research in Cameroon. Due to these low fat contents (<1%), cassava flour may have very low susceptibility to form a starch lipid complex or amylose lipid complex [33] which leads to low swelling capacity, solubility, and granule disruption [34]. Further, these low fat contents may facilitate to prepare low-fat food formulations such as soups and porridges.

Protein is an important macronutrient and a functional ingredient in food formulations. The protein contents of the five flour samples were ranged from 1.1% (MU51) to 1.7% (*Swarna*) which were not significantly different (*p* > 0.05) among five cassava varieties. However, the protein contents of tested cassava flours were significantly lower (*p* < 0.05) than those of commercial wheat flour (Table 1). Similar protein contents in cassava flour have been observed by Tambo et al. [28], Dudu et al. [4], and Klang et al. [30] which were 1.14%, 1.55%, and 1.6%, respectively. According to the findings of Oyeyinka et al. [35] and Abiodun et al. [36], the contents of protein, essential amino acids, and protein quality of cassava flour can be enhanced by the fermentation. Apart from the fermentation, fortification with protein rich sources [10] and formulation of composite flour with legume and cereal flour [9] have been proposed to improve the protein content and nutrition level of cassava flour. However, low protein content (<2%) of studied cassava flour may facilitate the preservation of color by avoiding the Maillard reaction which is observed in the extrusion process of potato flour [37].

### 3.3. Physicochemical Properties

#### 3.3.1. Hydrogen Cyanide (HCN)

**Figure 3:**
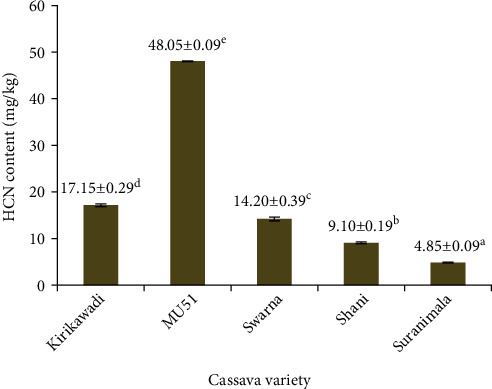
HCN content of cassava flours from five Sri Lankan cassava varieties. Values are expressed as mean ± standard deviation of three independent determinations. Different superscripts represent significantly different samples (*p* < 0.05).

The HCN is the most toxic compound that limits the use of cassava in foods. The recommended safety limit of HCN in edible cassava flour is 10 mg/kg [31]. The HCN contents of flours from *Suranimala* and *Shani* were lower than the levels recommended by WHO, indicating that these two varieties can be directly utilized in the food applications. However, MU51, *Swarna*, and *Kirikawadi* varieties contained significantly (*p* < 0.05) higher HCN contents (Figure 3), and minor modifications to the flour preparation process are required to reduce HCN content up to the acceptable level. The variations in HCN contents might be due to genotype, cultivar, and environmental conditions such as soil type, humidity, temperature, maturity level, nutritional status of the plant, climatic conditions during the harvesting time, application of inorganic fertilizers, and environmental pollution [38]. Wet fermentation, which facilitates the detoxification more than 94% [38], can be recommended to reduce the cyanide content and maximize the utilization of these three varieties.

#### 3.3.2. Color

The color gives the first sensation to the consumers in selecting a food product. The lightness of cassava flour from five varieties that ranged from 95.50 (*Swarna*) to 97.27 (MU51) was significantly higher (*p* < 0.05) than the lightness of commercial wheat flour (Table 2). Chisenga et al. [1] have observed little less lightness value from 93.65 to 94.69 for cassava flour. The average values of *a*∗ and *b*∗ of five cassava flour samples were significantly lower (*p* < 0.05) than *a*∗ and *b*∗ values of wheat flour. Falade et al. [39] have reported that cassava flour has high whiteness and lightness with low redness and yellowness. These color differences can be observed visually in flesh and flour, but not in starch [12]. The high value of *b*∗ in *Swarna* variety may be attributed by high carotene content. Gegios et al. [40] has stated that cassava varieties with colored flesh contained higher amount of carotene than varieties with white flesh. The color of cassava flour is important in foods such as ice creams, flavored beverages, desserts, cakes [41], strips [5], gruels [28, 30], gluten-free pasta [8], and gluten-free cookies [42]. Moreover, it is important in cassava starch industry [43].

#### 3.3.3. Total Starch

**Figure 4:**
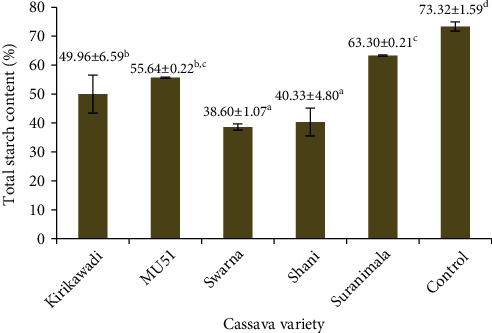
Starch contents of cassava flours from five Sri Lankan cassava varieties. Values are expressed as mean ± standard deviation of three independent determinations. Different superscripts represent significantly different samples (*p* < 0.05). Commercial wheat flour was used as the control.

Chemical analysis of starch revealed that starch contents of studied cassava flour vary from 38.60% (*Swarna*) to 63.30% (*Suranimala*) (Figure 4). The significant difference (*p* < 0.05) in starch contents between cassava flours could be due to genotype effect [44], maturity level [44], and agricultural practices [45]. Schmitz et al. [46] have observed low starch contents (17.28-35.37%) compared to the present results in ten Brazilian cassava varieties. However, Aryee et al. [47] have observed high starch contents (67.92-88.11%) in certain West African cassava varieties compared to the present results. Cassava starch is an important food ingredient, which has similar digestibility as rice, and taro [43] is widely used as a raw material in the food and nonfood product manufacturing industries. Out of five varieties tested, MU51 and *Suranimala* can be used in producing starch due to considerably higher starch contents. The functional properties of cassava starches may be improved via chemical, physical, biotechnological, and enzymatic modifications to improve the starch industry [43].

#### 3.3.4. Starch Granule Size and Shape

**Figure 5:**
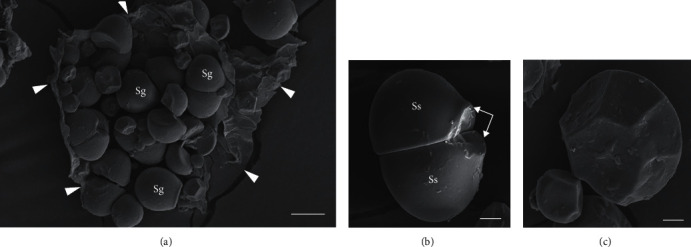
Scanning electron micrographs of starch granules observed in the flour extracted from Sri Lankan cassava starchy roots. (a) Clusters of starch granules (Sg) enclosed in parenchyma tissues (demarcation indicated by white arrowheads) (bar = 10 *μ*m). (b) A starch granule with smooth surface (SS). Note the circular shape with flat ends (white arrows) (bar = 4 *μ*m). (c) Starch granules with truncated shape. Note the size difference of the two granules (bar = 2 *μ*m).

**Figure 6:**
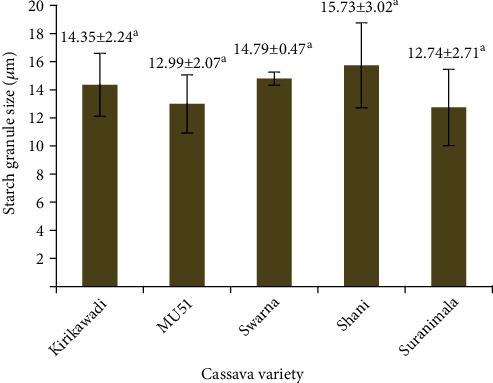
Starch granule size of cassava flours from five Sri Lankan cassava varieties. Values are expressed as mean ± standard deviation of three independent determinations. Different superscripts represent significantly different samples (*p* < 0.05).

Concerning SEM results, we observed that clusters of cassava starch granules had smooth surfaces and truncated shapes or circular shapes with flat surfaces on one face enclosed within the parenchyma tissues (Figure 5). The cassava starch granule sizes varied from 12.74 *μ*m (*Suranimala*) to 15.73 *μ*m (*Shani*), and results revealed that starch granule sizes of five cassava varieties were not significantly different (*p* > 0.05) (Figure 6). These findings were in agreement with Vasconcelos et al. [48] and Udoro et al. [49]. According to Zhang et al. [50], starch granules can be categorized as A type granules (>15 *μ*m), B type granules (5-15 *μ*m), and C type granules (<5 *μ*m). The present observations demonstrated that studied cassava varieties contain B type granules, which are suitable for modifications such as octenylsuccinylation to form pickering emulsifiers [51]. Moreover, small starch granules can facilitate competent hydration and swelling capacity, which leads to low gelatinization temperature [52].

### 3.4. Amylose and Amylopectin

**Figure 7:**
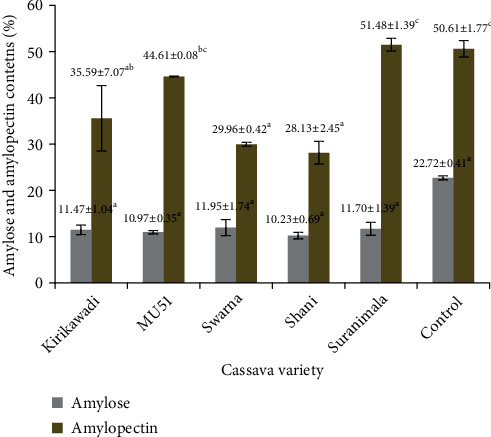
Amylose and amylopectin contents of cassava flours from five Sri Lankan cassava varieties. Values are expressed as mean ± standard deviation of three independent determinations. Different superscripts represent significantly different samples (*p* < 0.05). Commercial wheat flour was used as the control.

Results revealed that *Swarna* cassava flour contained the highest amylose content (11.95%). The flours of all five cassava varieties contained a comparable amount of amylose (10.23-11.95%), and they were significantly lower (*p* < 0.05) than the amylose content of wheat flour (22.72%) (Figure 7). The amylose contents of tested cassava flours were lower than the amylose contents of cassava flours of ten cassava varieties (14.80-22.78%) studied in Brazil [46], cassava flour (37-42%) in Cameroon [28], and commercial cassava flour (24.83%) in China [53]. This may be due to the climatic and varietal differences. The low amylose contents facilitate their starch stability in cold temperature [54]; thus, cassava flour from studied varieties may provide an attractive offer to produce modified starch for frozen and refrigerated foods. The major proportion of starch in five cassava varieties consistent with amylopectin was ranged from 28.13 to 51.48%. *Swarna* cassava flour showed the highest amylopectin content, which was approximately similar to commercial wheat flour. According the literature, the amylose and amylopectin ratio influences the hydration properties of starch granules. On the other hand, Vamadevan et al. [55] found that the length and four different forms of amylopectins, each with its own structural pattern and swelling capabilities, affect the starch gelatinization and retrogradation capabilities. Therefore, more research is needed to link the amylose and amylopectin ratio to the functional properties of flours from studied cassava varieties. Further, it has been reported that hydration properties of starch are influenced by granule architecture of native starch granules, availability of free hydroxyl groups in crystalline and amorphous areas, hydrophilic nature of starch granules, hydrothermal treatment, and chemical and enzymatic modifications other than the amylose and amylopectin ratio [56, 57].

### 3.5. Functional Properties

#### 3.5.1. Water Absorption Capacity (WAC)

Results showed that WACs of studied cassava flour samples varied from 159% (*Suranimala*) to 308% (*Kirikawadi*) (Table 3) and had higher WACs when compared with the commercial wheat flour. The WAC is an indication of the amount of water absorbed by flour under minimum water supply [58]. A greater level of starch crystal destruction [6] may attribute to higher WACs, and as mentioned beforehand granule architecture, amylose and amylopectin may influence the WACs of cassava flour. Godswill [59] has mentioned that polar amino residues with high affinity to water molecule also tend to support high WACs in cassava flour. High WACs (>100%) facilitate the addition of more water during the food processing and easy dough handling [60]. Therefore, flour from *Kirikawadi* may be the most suitable variety for the easy dough handling purpose in combination with wheat flour, and other four varieties may be suitable for foods such as porridges and gruels [9, 42].

#### 3.5.2. Oil Absorption Capacity (OAC)

The OACs of studied cassava flours were varied from 96.79% (MU51) to 118.83% (*Swarna*) and had lower OACs when compared with the commercial wheat flour (Table 3). In accordance with Godswill [59], low protein contents of cassava flour were reasoned to have low OACs as protein has positive relationship with OAC. The OAC is an indication of protein and fat interaction in food formulations [6]. Physical binding of oil by capillary attraction and hydrophobicity of proteins is an important phenomenon in oil absorption [58]. High OACs facilitate the flavor retention and improve the palatability of foods [60]. Therefore, flour from *Swarna* variety may be suitable to develop various food products where the high OAC is required such as minced meat formulations, meat analogues, extenders, and soups.

#### 3.5.3. Water Solubility Index (WSI) and Swelling Power (SP)

The highest WSI was observed in *Suranimala*, and it was significantly higher (*p* < 0.05) than the WSI of commercial wheat flour (Table 3). The highest SP was observed in MU51, and it was not significantly different (*p* > 0.05) from the SP of commercial wheat flour (Table 3). The WSI and SP are indicators of starch hydration [58]. They demonstrate the degree of interactions between the starch chains within both the amorphous and crystalline areas [10]. As mentioned previously, studied cassava flour contained low amylose contents. Low amount of amylose is an indication of lower degree of intermolecular associations. This may be due to the amorphous nature of amylose that facilitates rapid hydration than crystalline nature of amylopectin [61]. The WSI and SP are important to uniform food systems during food processing [10]. Moreover, WSI and SP values determine the textural, pasting, and thickening properties of starch-based food preparations. Flours from studied cassava varieties may be suitable to develop consistent dough, which may help to produce foods with good eating quality.

#### 3.5.4. Gelatinization Temperature (GT)

Results revealed that GT of five flours was not significantly different (*p* > 0.05) among the varieties but significantly higher (*p* < 0.05) than the GT of commercial wheat flour (Table 3). The studied cassava flour showed high GT though cassava flour contained low amylose contents compared to wheat flour. This may be due to the differences in tuber and cereal starches. However, it should be noted that amylose is not the only factor that influences starch functionality and GT of starch depends on different parameters including plant type, pH, salt concentration, amount of water, sugar, protein, and fat [59]. The GT of native cassava starch can be modified by processes such as acid treatments, acetylation, crosslinking, and gene manipulation techniques [62]. Gelatinization is important in the food industry such as manufacturing food additives where starch is used for thickening, stabilizing, or binding purpose. In terms of GT in food processing, lower gelatinization temperatures are better in foods containing heat-sensitive ingredients, and it could save processing time and energy consumption.

#### 3.5.5. Bulk Density (BD)

The porosity of food products is determined by BD. It depends on the initial moisture content, particle size, and starch content of flour [10]. The BDs of five flour samples were not significantly different (*p* > 0.05) and significantly lower (*p* < 0.05) than the BD of commercial wheat flour (Table 3). Klang et al. [30], Dereje et al. [58], and Anosike et al. [63] have mentioned that flours with BD lower than 1 g/mL can be used in manufacturing low-bulk weaning foods and high-energy foods. In addition, it facilitates easy storage, transport, and marketing due to low volume of packaging material requirement to store the flours [58].

#### 3.5.6. Emulsion Activity (EA) and Emulsion Stability (ES)

According to the present observations, *Kirikawadi*, MU51, and *Swarna* had significantly higher (*p* < 0.05) EA and ES values than commercial wheat flour (Table 3). As mentioned in the previous studies, there is a positive correlation between protein content and EA and ES. This statement does not agree with the present results as wheat flour showed low EA and ES although it contained high amount of protein. This may be due to the poor adsorption of wheat protein to the oil-water interface of the emulsion. Among studied varieties, flour from *Kirikawadi*, MU51, and *Swarna* may be useful in food products such as spreads, salad dressings, frozen desserts, frankfurter, sausage, and cakes due to their EA and ES.

#### 3.5.7. Antioxidant Properties

Higher prevalence of noncommunicable diseases has gained considerable attention of antioxidant properties of foods other than their nutritional composition. Phenolics are natural antioxidant compounds, which are capable of scavenging free radicals, inhibiting oxidases, activating antioxidant enzymes, and reducing metallic ions [64]. TPCs of five cassava flour samples were significantly different (*p* < 0.05) (Table 4) and ranged from 2.69 (*Kirikawadi*) to 4.44 (*Swarna*) mmol GAE/100 g dry weight. However, Lima et al. [65] have observed high content (36.25–56.44 mmol GAE/100 g dry weight) of extractable polyphenols in cassava after cooking. TFCs of five cassava flour samples were significantly different (*p* < 0.05) (Table 4) and ranged from 0.44 (*Suranimala*) to 0.60 (MU51) mmol QE/100 g dry weight. The DRSA of selected cassava varieties was ranged from 31% (*Suranimala)* to 72% (*Swarna)*, which was significantly (*p* < 0.05) the highest. The FRAP was ranged from 0.83 (*Kirikawadi*) to 0.93 (*Swarna*) mol GAE/100 g dry weight. Among tested varieties, only *Swarna* contained yellow-colored flesh. The highest amount of phenolic compounds was observed in *Swarna* relative to other four varieties. Therefore, high amounts of carotene content and phenolic compounds may be the reasons for the highest inhibition percentage in *Swarna*. The variation in the antioxidant content may be due to the varietal differences in cassava. The differences in genotypes highly influence the antioxidant capacity and phenolic content or root and tuber crops [66]. In addition, Heimler et al. [67] have observed that fertilizers influence the phytochemical content of plants. Considering antioxidant properties of *Swarna*, it may be suitable for functional food formulations to prevent oxidative stress-related diseases. However, further research studies are required to analyze antioxidant availability of the final products.

## 4. Conclusion

This study revealed that proximate composition and physicochemical, functional, and antioxidant properties of tested cassava flours were considerably affected by the varietal differences. The total starch contents of five varieties were lower than those of commercial wheat flour, and the major part of starch consisted of amylopectin. Flours of *Suranimala* and *Shani* varieties contained low amount of cyanide contents relative to the recommended levels. Water absorption capacity, oil absorption capacity, water solubility index, swelling power, emulsion activity, and emulsion stability of five flour types were significantly lower (*p* < 0.05) than those of wheat flour. Flour from *Swarna* variety demonstrated a strong antioxidant capacity and a high phenolic content compared to the other varieties. Flours from all cassava varieties contained acceptable functional properties, demonstrating the suitability to be utilized as an ingredient/raw material in different food formulations. Further, flours of these cassava varieties can be combined with the flours from other grains and legumes to develop functional flours for modern nutrimental applications.

## Figures and Tables

**Table 1:** Proximate composition of cassava flours from five Sri Lankan cassava varieties.

Chemical property	Cassava varieties					
	*Kirikawadi*	MU51	*Swarna*	*Shani*	*Suranimala*	Control
Moisture (%)	6.56 ± 0.29^b^	4.45 ± 0.39^a^	9.91 ± 0.28^c^	5.36 ± 0.01^a^	6.47 ± 0.00^b^	12.49 ± 0.39^d^
Ash (%)	1.05 ± 0.51^a^	1.12 ± 0.26^a^	1.69 ± 0.04^a,b^	1.01 ± 0.40^a^	2.06 ± 0.01^b^	1.52 ± 0.14^a,b^
Fat (%)	0.28 ± 0.01^a,b^	0.21 ± 0.06^a^	0.42 ± 0.03^a,b^	0.63 ± 0.04^b^	0.64 ± 0.12^b^	1.72 ± 0.16^c^
Protein (%)	1.18 ± 0.08^a^	1.09 ± 0.22^a^	1.70 ± 0.03^a^	1.16 ± 0.01^a^	1.30 ± 0.02^a^	8.28 ± 0.41^b^
Carbohydrate (%)	90.93	93.13	86.28	91.84	89.53	75.99

Values are expressed as mean ± standard deviation of three independent determinations. Different superscripts in a row represent significantly different samples (*p* < 0.05). Contents of carbohydrate were calculated by difference. Commercial wheat flour was used as the control.

**Table 2:** Color parameters of cassava flours from five Sri Lankan cassava varieties.

Color parameter	Cassava variety					
	*Kirikawadi*	MU51	*Swarna*	*Shani*	*Suranimala*	Control
Lightness (*L*∗)	95.77 ± 0.06^b^	97.27 ± 0.23^c^	95.50 ± 0.10^b^	96.60 ± 0.20^bc^	96.60 ± 0.17^bc^	91.00 ± 1.00^a^
Redness/greenness (*a*∗)	−0.23 ± 0.06^b^	−0.70 ± 0.20^b^	−0.40 ± 0.17^b^	−0.60 ± 0.00^b^	−0.50 ± 0.00^b^	1.84 ± 0.40^a^
Yellowness/blueness (*b*∗)	6.17 ± 0.12^b^	4.10 ± 0.10^a^	6.30 ± 0.10^b^	5.60 ± 0.36^b^	5.10 ± 0.20^a,b^	9.54 ± 1.01^c^

Values are expressed as mean ± standard deviation of three independent determinations. Different superscripts in a row represent significantly different samples (*p* < 0.05). Commercial wheat flour was used as the control.

**Table 3:** Functional properties of cassava flours from five Sri Lankan cassava varieties.

Character	Cassava variety					
	*Kirikawadi*	MU51	*Swarna*	*Shani*	*Suranimala*	Control
WAC (%)	308.25 ± 7.23^d^	275.18 ± 12.99^c,d^	214.43 ± 22.68^b^	261.30 ± 11.16^c^	159.72 ± 5.61^a^	142.97 ± 6.54^a^
OAC (%)	110.70 ± 0.68^a,b^	96.79 ± 2.95^a^	118.83 ± 6.98^b^	110.57 ± 10.56^a,b^	98.89 ± 4.44^a,b^	151.64 ± 4.94^c^
WSI (%)	2.72 ± 0.19^a,b^	1.92 ± 0.25^a^	2.17 ± 0.08^a,b^	2.97 ± 0.01^a,b^	4.08 ± 1.40^b^	1.95 ± 0.03^a^
SP (g/g)	6.79 ± 0.10^a^	16.43 ± 0.99^c^	8.09 ± 0.42^a^	10.80 ± 0.33^b^	10.40 ± 0.39^b^	17.20 ± 0.36^c^
GT (°C)	68.00 ± 1.00^b^	67.33 ± 2.08^b^	69.33 ± 0.58^b^	68.33 ± 0.58^b^	67.67 ± 1.53^b^	60.08 ± 0.09^a^
BD (g/mL)	0.497 ± 0.022^a^	0.489 ± 0.048^a^	0.497 ± 0.018^a^	0.501 ± 0.019^a^	0.486 ± 0.014^a^	0.753 ± 0.01^b^
EA (%)	52.54 ± 0.54^a,b^	60.86 ± 5.04^b^	53.52 ± 0.00^a,b^	46.64 ± 6.99^a^	46.54 ± 2.34^a^	43.90 ± 2.81^a^
ES (%)	45.72 ± 1.27^a,b^	48.82 ± 0.55^b^	46.85 ± 0.53^a,b^	43.15 ± 0.41^a^	43.39 ± 1.32^a^	43.19 ± 1.84^a^

Values are expressed as mean ± standard deviation of three independent determinations. Different superscripts in a row represent significantly different samples (*p* < 0.05). WAC: water absorption capacity; OAC: oil absorption capacity; WSI: water solubility index; SP: swelling power; LGC: least gelation concentration; GT: gelatinization temperature; BD: bulk density; EA: emulsion activity; and ES: emulsion stability. Wheat flour was used as the control.

**Table 4:** Antioxidant properties of cassava flours from five Sri Lankan cassava varieties.

Parameter	Cassava variety				
	*Kirikawadi*	MU51	*Swarna*	*Shani*	*Suranimala*
TPC (mmol GAE/100 g dry weight)	2.69 ± 0.21^a^	2.75 ± 0.26^a^	4.44 ± 0.06^c^	3.60 ± 0.03^b^	3.75 ± 0.07^b^
TFC (mmol QE/100 g dry weight)	0.52 ± 0.004^c^	0.60 ± 0.001^d^	0.47 ± 0.001^b^	0.47 ± 0.01^b^	0.44 ± 0.002^a^
DRSA (% inhibition)	45.67 ± 6.34^a^	32.97 ± 0.11^a^	72.01 ± 0.89^b^	42.54 ± 3.87^a^	31.45 ± 4.89^a^
FRAP (mol GAE/100 g dry weight)	0.83 ± 0.16^a^	0.87 ± 0.31^ab^	0.93 ± 0.21^ab^	0.87 ± 0.49^ab^	0.90 ± 0.19^b^

Values are expressed as mean ± standard deviation of three independent determinations. Different superscripts in a row represent significantly different samples (*p* < 0.05). TPC: total phenolic content; TFC: total flavonoid content; DRSA: DPPH radical scavenging activity; FRAP: ferric reducing antioxidant power.

## Data Availability

Data are available on request.

## References

[1] Chisenga S. M., Workneh T. S., Bultosa G., Alimi B. A. (2019). Progress in research and applications of cassava flour and starch: a review. *Journal of Food Science and Technology*.

[2] Bouniol A., Adinsi L., Padonou S. W. (2021). Rheological and textural properties of lafun, a stiff dough, from improved cassava varieties. *International Journal of Food Science and Technology*.

[3] Ajani R., Oboh G., Adefegha S. A., Nwokocha K. E., Akindahunsi A. A. (2020). Sensory attributes, nutritional qualities, and glycemic indices of bread blends produced from cocoa powder flavored yellow-fleshed cassava-wheat composite flours. *Journal of Food Processing and Preservation*.

[4] Dudu O. E., Ma Y., Adelekan A., Oyedeji A. B., Oyeyinka S. A., Ogungbemi J. W. (2020). Bread-making potential of heat-moisture treated cassava flour-additive complexes. *International Journal of Biological Macromolecules*.

[5] Dada T. A., Barber L. I., Ngoma L., Mwanza M. (2018). Formulation, sensory evaluation, proximate composition and storage stability of cassava strips produced from the composite flour of cassava and cowpea. *Food Science and Nutrition*.

[6] Lu H., Guo L., Zhang L. (2020). Study on quality characteristics of cassava flour and cassava flour short biscuits. *Food Science and Nutrition*.

[7] Nilusha R. A. T., Jayasinghe J. M. J. K., Perera O. D. A. N., Perera P. I. P. (2019). Development of pasta products with nonconventional ingredients and their effect on selected quality characteristics: a brief overview. *International Journal of Food Science*.

[8] Rachman A., Brennan M. A., Morton J., Brennan C. S. (2020). Gluten-free pasta production from banana and cassava flours with egg white protein and soy protein addition. *International Journal of Food Science and Technology*.

[9] Onyango C., Luvitaa S. K., Unbehend G., Haase N. (2020). Nutrient composition, sensory attributes and starch digestibility of cassava porridge modified with hydrothermally-treated finger millet. *Journal of Agriculture and Food Research*.

[10] Otondi E. A., Nduko J. M., Omwamba M. (2020). Physico-chemical properties of extruded cassava-chia seed instant flour. *Journal of Agriculture and Food Research*.

[11] Pérez-Vergara L. D., Cifuentes M. T., Franco A. P., Pérez-Cervera C. E., Andrade-Pizarro R. D. (2020). Development and characterization of edible films based on native cassava starch, beeswax, and propolis. *NFS Journal*.

[12] Ayetigbo O., Latif S., Abass A., Müller J. (2019). Preparation, optimization and characterization of foam from white-flesh and yellow-flesh cassava (*Manihot esculenta*) for powder production. *Food Hydrocolloids*.

[13] Bala A., Gul K., Riar C. S. (2015). Functional and sensory properties of cookies prepared from wheat flour supplemented with cassava and water chestnut flours. *Cogent Food and Agriculture*.

[14] Bradbury J. H. (2006). Simple wetting method to reduce cyanogen content of cassava flour. *Journal of Food Composition and Analysis*.

[15] AOAC (2016). *Official Methods of Analysis of AOAC International*.

[16] Sompong R., Siebenhandl-Ehn S., Linsberger-Martin G., Berghofer E. (2011). Physicochemical and antioxidative properties of red and black rice varieties from Thailand, China and Sri Lanka. *Food Chemistry*.

[17] Alamu E. O., Maziya-Dixon B., Dixon A. G. (2017). Evaluation of proximate composition and pasting properties of high quality cassava flour (HQCF) from cassava genotypes (*Manihot esculenta Crantz*) of *β*-carotene-enriched roots. *LWT Food Science and Technology*.

[18] Zhou W., Yang J., Hong Y. (2015). Impact of amylose content on starch physicochemical properties in transgenic sweet potato. *Carbohydrate Polymers*.

[19] Sosulski F. W. M. O., Garratt M. D., Slimkard A. E. (1976). Functional properties of ten legume flours. *Canadian Institute of Food Science and Technology Journal*.

[20] Leach W., McCowen D., Schoch T. J. (1959). Structure of the starch granule. I. Swelling and solubility patterns of various starches. *Cereal Chemistry*.

[21] Chandra S., Singh S., Kumari D. (2014). Evaluation of functional properties of composite flours and sensorial attributes of composite flour biscuits. *Journal of Food Science and Technology*.

[22] Chandrasekara A., Josheph Kumar T. (2016). Roots and tuber crops as functional foods: a review on phytochemical constituents and their potential health benefits. *International Journal of Food Science*.

[23] Singleton V. L., Orthofer R., Lamuela-Raventós R. M. (1999). [14] Analysis of total phenols and other oxidation substrates and antioxidants by means of Folin-Ciocalteu reagent. *Methods in Enzymology*.

[24] Kalita P., Tapan B. K., Pal T. K., Kalita R. (2013). Estimation of total flavonoids content (TFC) and anti-oxidant activities of methanolic whole plant extract of *Biophytum sensitivum* Linn. *Journal of Drug Delivery and Therapeutics*.

[25] Kourouma V., Mu T. H., Zhang M., Sun H. N. (2019). Effects of cooking process on carotenoids and antioxidant activity of orange-fleshed sweet potato. *International Journal of Food Science Technology*.

[26] Davies-Hoes L. D., Scanlon M. G., Girgih A. T., Aluko R. E. (2017). Effect of pea flours with different particle sizes on antioxidant activity in pan breads. *Cereal Chemistry*.

[27] DOA (2019). *Department of Agriculture Horticulture, Crop Research and Development Institute (HORDI)*.

[28] Tambo Tene S., Klang J. M., Ndomou Houketchang S. C., Teboukeu Boungo G., Womeni H. M. (2019). Characterization of corn, cassava, and commercial flours: use of amylase-rich flours of germinated corn and sweet potato in the reduction of the consistency of the gruels made from these flours-influence on the nutritional and energy value. *Food Science and Nutrition*.

[29] Maziya-Dixon B., Alamu E. O., Popoola I. O., Yomeni M. (2017). Nutritional and sensory properties: snack food made from high-quality cassava flour and legume blend. *Food Science and Nutrition*.

[30] Klang J. M., Tambo Tene S., Matueno Kamdem F. E., Teboukeu Boungo G., Womeni H. M. (2020). Optimization using response surface methodology (RSM) of the energy density of flour-based gruels of sweet cassava (*Manihot esculenta* Crantz) flour: effect of the addition of two new sprouted rice varieties produced under optimal conditions (*Nerica 3* and *Nerica L56*). *NFS Journal*.

[31] Codex Alimentarius Commission (1995). *Edible Cassava Flour (CODEX STAN 176-1989 (Rev.1–1995))*.

[32] Bolaji O. T., Kamoru M. A., Adeyeye S. A. O. (2021). Quality evaluation and physico-chemical properties of blends of fermented cassava flour (lafun) and pigeon pea flour. *Scientific African*.

[33] Wheatley C. C., Chuzel G., Zakhia N. (2003). Cassava–uses as feedstock. *Encyclopedia of Food Sciences and Nutrition*.

[34] Huang Q., Chen X., Wang S., Zhu J. (2020). Amylose–lipid complex. *Starch Structure, Functionality and Application in Foods*.

[35] Oyeyinka S. A., Ayinla S. O., Sanusi C. T. (2020). Chemical and physicochemical properties of fermented flour from refrigerated cassava root and sensory properties of its cooked paste. *Journal of Food Processing and Preservation*.

[36] Abiodun O. A., Ayano B., Amanyunose A. A. (2020). Effect of fermentation periods and storage on the chemical and physicochemical properties of biofortified cassava gari. *Journal of Food Processing and Preservation*.

[37] Ganjyal G. M. (2020). *Extrusion Cooking: Cereal Grains Processing*.

[38] Njankouo Ndam Y., Mounjouenpou P., Kansci G. (2019). Influence of cultivars and processing methods on the cyanide contents of cassava (*Manihot esculenta Crantz*) and its traditional food products. *Scientific African*.

[39] Falade K. O., Ibanga-Bamijoko B., Ayetigbo O. E. (2019). Comparing properties of starch and flour of yellow-flesh cassava cultivars and effects of modifications on properties of their starch. *Journal of Food Measurement and Characterization*.

[40] Gegios A., Amthor R., Maziya-Dixon B. (2010). Children consuming cassava as a staple food are at risk for inadequate zinc, iron, and vitamin A intake. *Plant Foods for Human Nutrition*.

[41] Vega O., Carvajal L. M., Rodríguez F. (2018). Effect of thermal pretreatments and cooking characteristics on physicochemical, rheological, and sensorial properties of food products based on cassava (*Manihot esculenta* Crantz). *Journal of Food Process Engineering*.

[42] Giri N. A., Sakhale B. K. (2021). Effects of incorporation of orange-fleshed sweet potato flour on physicochemical, nutritional, functional, microbial, and sensory characteristics of gluten-free cookies. *Journal of Food Processing and Preservation*.

[43] Li Y., Li C., Gu Z., Hong Y., Cheng L., Li Z. (2017). Effect of modification with 1,4-*α*-glucan branching enzyme on the rheological properties of cassava starch. *International Journal of Biological Macromolecules*.

[44] Benesi I. R. M., Labuschagne M. T., Herselman L., Mahungu N. M., Saka J. K. (2008). The effect of genotype, location and season on cassava starch extraction. *Euphytica*.

[45] Luchese C. L., Spada J. C., Tessaro I. C. (2017). Starch content affects physicochemical properties of corn and cassava starch-based films. *Industrial Crops and Products*.

[46] Schmitz G. J. H., de Magalhães Andrade J., Valle T. L., Labate C. A., Do Nascimento J. R. O. (2016). Comparative proteome analysis of the tuberous roots of six cassava (*Manihot esculenta*) varieties reveals proteins related to phenotypic traits. *Journal of Agricultural and Food Chemistry*.

[47] Aryee F. N. A., Oduro I., Ellis W. O., Afuakwa J. J. (2006). The physicochemical properties of flour samples from the roots of 31 varieties of cassava. *Food Control*.

[48] Vasconcelos L. M., Brito A. C., Carmo C. D., Oliveira P. H. G. A., Oliveira E. J. (2017). Phenotypic diversity of starch granules in cassava germplasm. *Genetics and Molecular Research*.

[49] Udoro E. O., Anyasi T. A., Jideani A. I. O. (2020). Characterization of the root and flour of South African Manihot esculenta Crantz landraces and their potential end-use properties. *International Journal of Food Properties*.

[50] Zhang L., Zhao Y., Hu W. (2018). Multi-scale structures of cassava and potato starch fractions varying in granule size. *Carbohydrate Polymers*.

[51] Li S., Zhang B., Li C., Fu X., Huang Q. (2020). Pickering emulsion gel stabilized by octenylsuccinate quinoa starch granule as lutein carrier: role of the gel network. *Food Chemistry*.

[52] Nawaz A., Ali S. W., Irshad S. (2020). Effect of peeling and unpeeling on yield, chemical structure, morphology and pasting properties of starch extracted from three diverse potato cultivars of Pakistan. *International Journal of Food Science and Technology*.

[53] Dudu O. E., Li L., Oyedeji A. B., Oyeyinka S. A., Ma Y. (2019). Structural and functional characteristics of optimised dry-heat-moisture treated cassava flour and starch. *International Journal of Biological Macromolecules*.

[54] Hsieh C. F., Liu W., Whaley J. K., Shi Y. C. (2019). Structure, properties, and potential applications of waxy tapioca starches - a review. *Trends in Food Science and Technology*.

[55] Vamadevan V., Bertoft E. (2020). Observations on the impact of amylopectin and amylose structure on the swelling of starch granules. *Food Hydrocolloids*.

[56] Goldstein A., Annor G., Vamadevan V. (2017). Influence of diurnal photosynthetic activity on the morphology, structure, and thermal properties of normal and waxy barley starch. *International Journal of Biological Macromolecules*.

[57] Alvani K., Qi X., Tester R. F. (2012). Gelatinisation properties of native and annealed potato starches. *Starch-Stärke*.

[58] Dereje B., Girma A., Mamo D., Chalchisa T. (2020). Functional properties of sweet potato flour and its role in product development: a review. *International Journal of Food Properties*.

[59] Godswill A. C. (2019). Proximate composition and functional properties of different grain flour composites for industrial applications. *International Journal of Food Sciences*.

[60] Jisha S., Sheriff J. T., Padmaja G. (2010). Nutritional, functional and physical properties of extrudates from blends of cassava flour with cereal and legume flours. *International Journal of Food Properties*.

[61] Kusumayanti H., Handayani N. A., Santosa H. (2015). Swelling power and water solubility of cassava and sweet potatoes flour. *Procedia Environmental Sciences*.

[62] Mason W. R. (2009). Starch use in foods. *Starch*.

[63] Anosike F. C., Nwagu K. E., Nwalo N. F. (2020). Functional and pasting properties of fortified complementary foods formulated from maize (*Zea mays*) and African yam bean (*Sphenostylis stenocarpa*) flours. *Legume Science*.

[64] Adefegha S. A. (2018). Impact of pasting on starch composition, estimated glycemic index, phenolic constituents, antioxidant activities and antidiabetic properties of flour produced from cocoyam (*Colocasia esculenta*) corm. *Journal of Food Biochemistry*.

[65] Lima E. C., Feijo M. B., Freitas M. C., Santos E. R., Sabaa-Srur A. U., Moura L. S. (2013). Sensorial evolution of cassava flour (*Manihot* esculenta Crantz) added to protein concentrate cassava leaves. *Food Science and Nutrition*.

[66] Grace M. H., Yousef G. G., Gustafson S. J., Truong V. D., Yencho G. C., Lila M. A. (2014). Phytochemical changes in phenolics, anthocyanins, ascorbic acid, and carotenoids associated with sweetpotato storage and impacts on bioactive properties. *Food Chemistry*.

[67] Heimler D., Romani A., Ieri F. (2017). Plant polyphenol content, soil fertilization and agricultural management: a review. *European Food Research and Technology*.

